# Supplementary figures and images for: Fine-scale mapping of chromosome 9q22.33 identifies candidate causal variant in ovarian cancer
Source: PeerJ. 2024 Feb 14;12:e16918. doi: 10.7717/peerj.16918 (PMC10874173; doi:10.7717/peerj.16918)

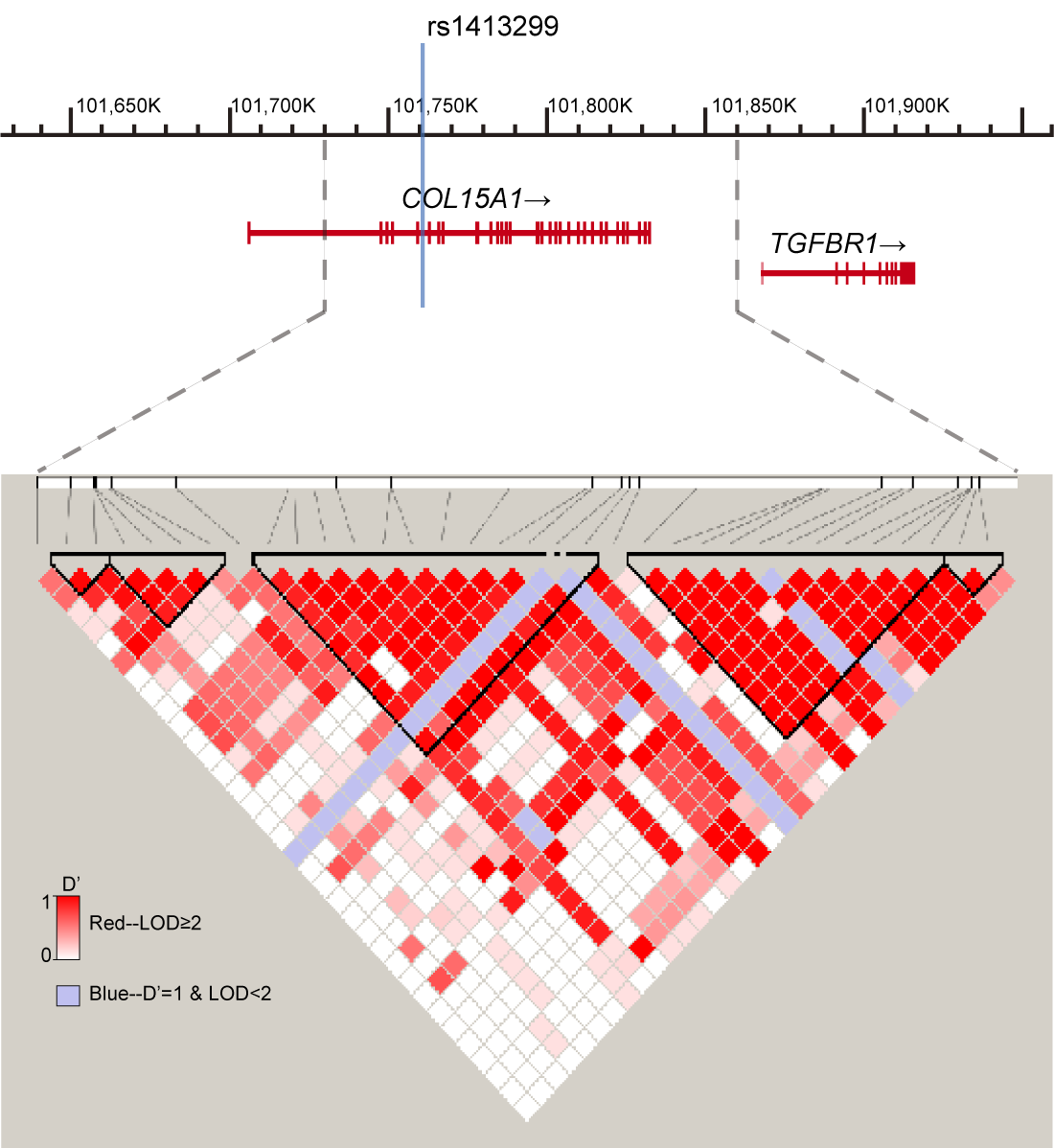

Supplement: Supplemental Information 1 — The high linkage disequilibrium between two variants is marked as a red square and low LD is marked as white. [file peerj-12-16918-s001.png]

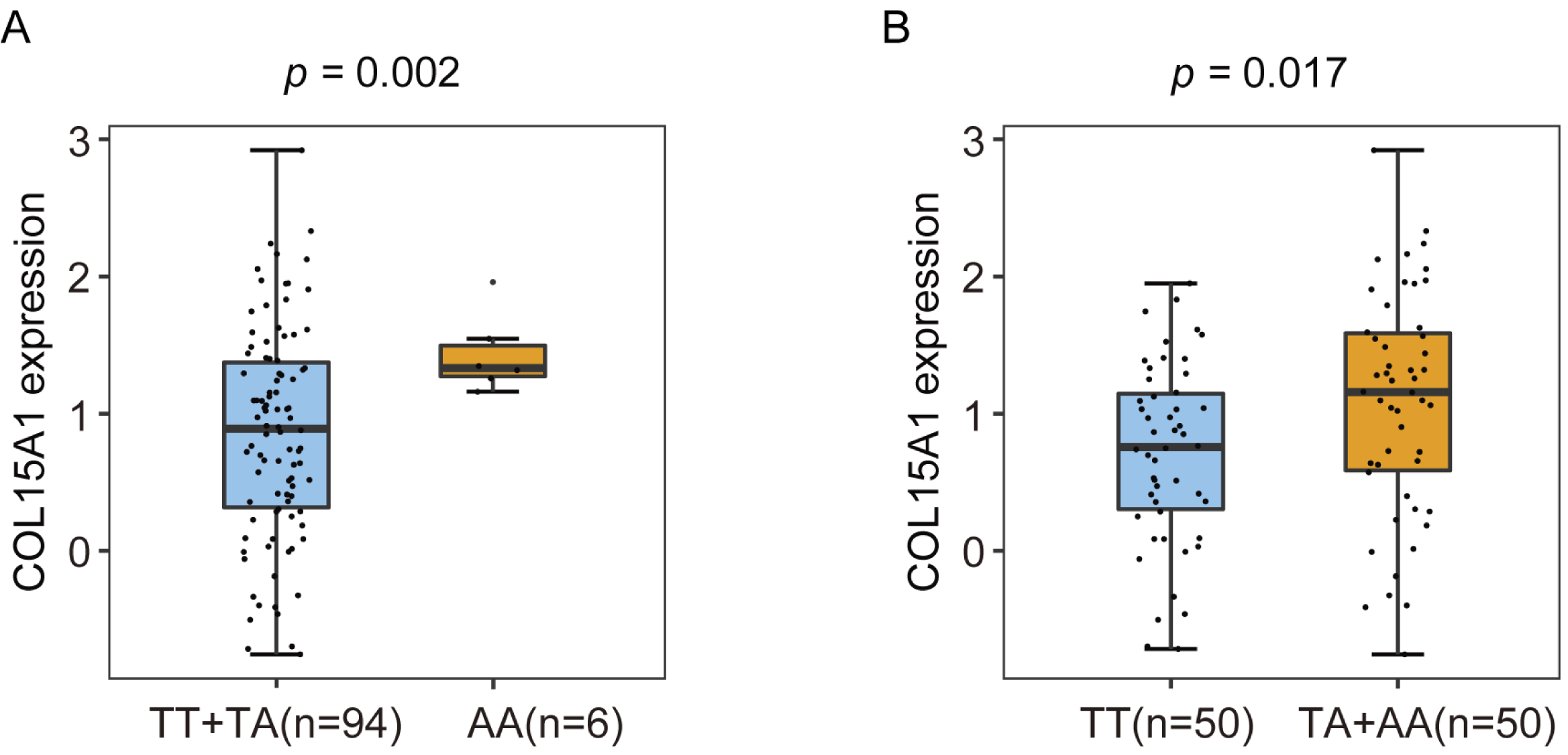

Supplement: Supplemental Information 2 [file peerj-12-16918-s002.png]
