# Supplementary material for: Fine-scale mapping of chromosome 9q22.33 identifies candidate causal variant in ovarian cancer
Source: PeerJ. 2024 Feb 14;12:e16918. doi: 10.7717/peerj.16918 (PMC10874173; doi:10.7717/peerj.16918)
Supplement: Supplemental Information 3 [file peerj-12-16918-s003.docx]

**Supplementary Table S1.** Primers used in current study.

| Locus | Primers (5'-3') |
| --- | --- |
| rs1889268（A） | F: CTTCCTTAAGTTCTATATCCCATCAAGCATC-biotin  R: GATGCTTGATGGGATATAGAACTTAAGGAAG |
| rs1889268（G） | F: CTTCCTTAAGTTCTACATCCCATCAAGCATC-biotin  R: GATGCTTGATGGGATGTAGAACTTAAGGAAG |
| rs7027650（A） | F: AGTTCTATGCAGAACATTTTTTTCTAAGTGG-biotin  R: CCACTTAGAAAAAAATGTTCTGCATAGAACT |
| rs7027650（T） | F: AGTTCTATGCAGAACTTTTTTTTCTAAGTGG-biotin  R: CCACTTAGAAAAAAAAGTTCTGCATAGAACT |
