# Supplementary material for: Fine-scale mapping of chromosome 9q22.33 identifies candidate causal variant in ovarian cancer
Source: PeerJ. 2024 Feb 14;12:e16918. doi: 10.7717/peerj.16918 (PMC10874173; doi:10.7717/peerj.16918)
Supplement: Supplemental Information 4 — MAF, Minor allele frequency; SNP, singlenucleotide polymorphism. a Position is GRCh37. b Major allele/minor allele. c r2 of linkage disequilibrium between variants with rs1413299. [file peerj-12-16918-s004.docx]

**Supplementary Table S2** Candidate causal variants selected for validation study from target sequencing.

| SNP | Chr. | Position^a^ | Allele^b^ | r^2^ ^c^ | MAF |
| --- | --- | --- | --- | --- | --- |
| rs4743305 | chr9 | 101760026 | C/T | 0.877 | 0.41 |
| rs7021675 | chr9 | 101752965 | G/A | 0.779 | 0.415 |
| rs1572136 | chr9 | 101740604 | C/G | 0.768 | 0.402 |
| rs10988451 | chr9 | 101741666 | A/G | 0.749 | 0.407 |

MAF, Minor allele frequency; SNP, singlenucleotide polymorphism.

^a^ Position is GRCh37.

^b^ Major allele/minor allele.

^c^ r^2^ of linkage disequilibrium between variants with rs1413299.
