# Supplementary material for: Fine-scale mapping of chromosome 9q22.33 identifies candidate causal variant in ovarian cancer
Source: PeerJ. 2024 Feb 14;12:e16918. doi: 10.7717/peerj.16918 (PMC10874173; doi:10.7717/peerj.16918)
Supplement: Supplemental Information 5 — GWAS, Genome-wide association study; MAF, minor allele frequencey; OR, odds ratio; SNP, single nucleotide polymorphism. a GWAS stage I in Han Chinese (1,172 controls/1,044 cases); genotyping using Illumina HumanOmniZhongHua-8 BeadChip. b Position is GRCh37. c Minor allele/major allele. d P value of association analysis using logistic regression adjusted for age and first three principal components of population stratification. [file peerj-12-16918-s005.docx]

**Supplementary Table S3** Candidate causal variants selected for validation study from the discovery stage of previous GWAS study^a^.

| SNP | Chr. | Position^b^ | Allele^c^ | MAF | OR | *P*^d^ |
| --- | --- | --- | --- | --- | --- | --- |
| rs1889268 | chr9 | 101767961 | T/C | 0.33 | 1.27 | 2.73E-04 |
| rs10819587 | chr9 | 101781301 | A/G | 0.21 | 1.30 | 4.79E-04 |
| rs73503719 | chr9 | 101768847 | A/G | 0.43 | 1.22 | 1.33E-03 |
| rs7031588 | chr9 | 101822302 | C/T | 0.42 | 1.22 | 1.57E-03 |
| rs1413298 | chr9 | 101823373 | A/G | 0.40 | 1.20 | 3.20E-03 |

GWAS, Genome-wide association study; MAF, minor allele frequencey; OR, odds ratio; SNP, single nucleotide polymorphism.

^a^ GWAS stage I in Han Chinese (1,172 controls/1,044 cases); genotyping using Illumina HumanOmniZhongHua-8 BeadChip.

^b^ Position is GRCh37.

^c^ Minor allele/major allele.

^d^ *P* value of association analysis using logistic regression adjusted for age and first three principal components of population stratification.
