# Supplementary material for: Fine-scale mapping of chromosome 9q22.33 identifies candidate causal variant in ovarian cancer
Source: PeerJ. 2024 Feb 14;12:e16918. doi: 10.7717/peerj.16918 (PMC10874173; doi:10.7717/peerj.16918)
Supplement: Supplemental Information 6 — a Position is GRCh37. b Risk allele/other allele. c r2 of linkage disequilibrium between variants with rs1413299. [file peerj-12-16918-s006.docx]

**Supplementary Table S4** Candidate causal variants selected for validation study through SNAP (high LD with rs1413299).

| SNP | Chr. | Position^a^ | Allele^b^ | r^2^ ^c^ |
| --- | --- | --- | --- | --- |
| rs7027650 | chr9 | 101741969 | T/A | 0.792 |
| rs10988451 | chr9 | 101741666 | G/A | 0.789 |

^a^ Position is GRCh37.

^b^ Risk allele/other allele.

^c^ r^2^ of linkage disequilibrium between variants with rs1413299.
