# Supplementary material for: Fine-scale mapping of chromosome 9q22.33 identifies candidate causal variant in ovarian cancer
Source: PeerJ. 2024 Feb 14;12:e16918. doi: 10.7717/peerj.16918 (PMC10874173; doi:10.7717/peerj.16918)
Supplement: Supplemental Information 7 — a: mean ±s.d. [file peerj-12-16918-s007.docx]

**Supplementary Table S5** Demographic characteristics of the participants.

|  | Control | Case_all | Serous | Mucinous | Endometrioid | Other |
| --- | --- | --- | --- | --- | --- | --- |
| N | 1591 | 1099 | 507 | 100 | 148 | 344 |
| Age^a^ | 52.13±12.53 | 52.67±11.99 | 52.75±11.66 | 48.04±14.82 | 53.25±11.57 | 53.67±11.46 |

a: mean ± s.d.
