# Supplementary material for: Fine-scale mapping of chromosome 9q22.33 identifies candidate causal variant in ovarian cancer
Source: PeerJ. 2024 Feb 14;12:e16918. doi: 10.7717/peerj.16918 (PMC10874173; doi:10.7717/peerj.16918)
Supplement: Supplemental Information 8 — CI, confidence interval; OR, odds ratio; RAF, risk allele frequency; SNP, single nucleotide polymorphism. a Position is given with respect to genome build 37. b Risk allele/other allele. [file peerj-12-16918-s008.docx]

**Supplementary Table S6**. Association results of ten SNPs at 9q22.33 in validation study.

| SNP | Position^a^ | Allele^b^ | RAF | OR(95%CI) | *P* |
| --- | --- | --- | --- | --- | --- |
| rs1572136 | 101740604 | G/C | 0.44 | 1.07(0.96-1.18) | 2.18E-01 |
| rs10988451 | 101741666 | G/A | 0.44 | 1.08(0.96-1.19) | 2.00E-01 |
| rs7027650 | 101741969 | T/A | 0.57 | 1.39(1.24-1.55) | 1.91E-07 |
| rs7021675 | 101752965 | A/G | 0.44 | 1.06(0.94-1.17) | 3.40E-01 |
| rs4743305 | 101760026 | T/C | 0.42 | 1.10(0.98-1.21) | 1.11E-01 |
| rs1889268 | 101767961 | T/C | 0.32 | 1.13(1.02-1.25) | 3.71E-02 |
| rs73503719 | 101768847 | A/G | 0.43 | 1.03(0.92-1.13) | 6.56E-01 |
| rs10819587 | 101781301 | A/G | 0.21 | 1.12(0.98-1.26) | 1.03E-01 |
| rs7031588 | 101822302 | C/T | 0.43 | 1.01(0.90-1.13) | 8.05E-01 |
| rs1413298 | 101823373 | A/G | 0.39 | 1.09(0.98-1.21) | 1.18E-01 |

CI, confidence interval;OR, odds ratio; RAF, risk allele frequency; SNP,singlenucleotide polymorphism.

^a^ Position is given with respect to genome build 37.

^b^ Risk allele/other allele.
