# Supplementary material for: Fine-scale mapping of chromosome 9q22.33 identifies candidate causal variant in ovarian cancer
Source: PeerJ. 2024 Feb 14;12:e16918. doi: 10.7717/peerj.16918 (PMC10874173; doi:10.7717/peerj.16918)
Supplement: Supplemental Information 9 — CI, confidence interval; OR, odds ratio; RAF, risk allele frequency; SNP, single nucleotide polymorphism. a Position is given with respect to genome build 37. b Risk allele/other allele [file peerj-12-16918-s009.docx]

**Supplementary Table S7** Association results of ten SNPs stratified by histologic subtypes in validation study.

| Histotype | SNP | Position ^a^ | Allele^b^ | RAF | OR(95%CI) | *P* |
| --- | --- | --- | --- | --- | --- | --- |
| Serous | rs1572136 | 101740604 | G/C | 0.44 | 1.15(1.01-1.29) | 5.41E-02 |
|  | rs10988451 | 101741666 | G/A | 0.44 | 1.15(1.01-1.30) | 5.18E-02 |
|  | rs7027650 | 101741969 | T/A | 0.55 | 1.30(1.12-1.50) | 1.33E-03 |
|  | rs7021675 | 101752965 | A/G | 0.44 | 1.11(0.97-1.26) | 1.40E-01 |
|  | rs4743305 | 101760026 | T/C | 0.42 | 1.14(0.99-1.29) | 7.83E-02 |
|  | rs1889268 | 101767961 | T/C | 0.32 | 1.17(1.01-1.32) | 4.99E-02 |
|  | rs73503719 | 101768847 | A/G | 0.43 | 1.01(0.87-1.15) | 8.69E-01 |
|  | rs10819587 | 101781301 | A/G | 0.21 | 1.15(0.98-1.33) | 1.11E-01 |
|  | rs7031588 | 101822302 | C/T | 0.58 | 1.10(0.95-1.27) | 2.03E-01 |
|  | rs1413298 | 101823373 | A/G | 0.62 | 1.01(0.87-1.17) | 8.86E-01 |
| Endometrioid | rs1572136 | 101740604 | G/C | 0.43 | 1.05(0.81-1.29) | 7.13E-01 |
|  | rs10988451 | 101741666 | G/A | 0.43 | 1.07(0.82-1.31) | 5.99E-01 |
|  | rs7027650 | 101741969 | T/A | 0.55 | 1.94(1.49-2.52) | 5.57E-06 |
|  | rs7021675 | 101752965 | A/G | 0.43 | 1.04(0.80-1.29) | 7.45E-01 |
|  | rs4743305 | 101760026 | T/C | 0.41 | 1.10(0.85-1.34) | 4.63E-01 |
|  | rs1889268 | 101767961 | T/C | 0.31 | 1.03(0.77-1.28) | 8.48E-01 |
|  | rs73503719 | 101768847 | A/G | 0.58 | 1.11(0.87-1.42) | 4.12E-01 |
|  | rs10819587 | 101781301 | A/G | 0.80 | 1.13(0.83-1.55) | 4.34E-01 |
|  | rs7031588 | 101822302 | C/T | 0.43 | 1.26(1.02-1.51) | 6.38E-02 |
|  | rs1413298 | 101823373 | A/G | 0.39 | 1.39(1.14-1.63) | 8.72E-03 |
| Mucinous | rs1572136 | 101740604 | G/C | 0.57 | 1.13(0.84-1.52) | 4.44E-01 |
|  | rs10988451 | 101741666 | G/A | 0.57 | 1.10(0.82-1.48) | 5.49E-01 |
|  | rs7027650 | 101741969 | T/A | 0.54 | 1.75(1.29-2.39) | 1.27E-03 |
|  | rs7021675 | 101752965 | A/G | 0.57 | 1.08(0.80-1.45) | 6.30E-01 |
|  | rs4743305 | 101760026 | T/C | 0.59 | 1.01(0.75-1.37) | 8.94E-01 |
|  | rs1889268 | 101767961 | T/C | 0.31 | 1.09(0.78-1.41) | 5.72E-01 |
|  | rs73503719 | 101768847 | A/G | 0.43 | 1.13(0.84-1.41) | 4.15E-01 |
|  | rs10819587 | 101781301 | A/G | 0.20 | 1.17(0.81-1.53) | 4.01E-01 |
|  | rs7031588 | 101822302 | C/T | 0.43 | 1.06(0.76-1.35) | 7.14E-01 |
|  | rs1413298 | 101823373 | A/G | 0.38 | 1.09(0.79-1.38) | 5.78E-01 |
| Other | rs1572136 | 101740604 | G/C | 0.43 | 1.03(0.86-1.20) | 7.24E-01 |
|  | rs10988451 | 101741666 | G/A | 0.43 | 1.02(0.85-1.20) | 7.84E-01 |
|  | rs7027650 | 101741969 | T/A | 0.54 | 1.24(1.04-1.48) | 2.59E-02 |
|  | rs7021675 | 101752965 | A/G | 0.43 | 1.02(0.85-1.19) | 8.21E-01 |
|  | rs4743305 | 101760026 | T/C | 0.41 | 1.07(0.90-1.24) | 4.30E-01 |
|  | rs1889268 | 101767961 | T/C | 0.31 | 1.15(0.97-1.33) | 1.26E-01 |
|  | rs73503719 | 101768847 | A/G | 0.43 | 1.07(0.90-1.23) | 4.23E-01 |
|  | rs10819587 | 101781301 | A/G | 0.21 | 1.17(0.96-1.38) | 1.33E-01 |
|  | rs7031588 | 101822302 | C/T | 0.43 | 1.07(0.90-1.24) | 4.49E-01 |
|  | rs1413298 | 101823373 | A/G | 0.39 | 1.14(0.97-1.31) | 1.27E-01 |

CI, confidence interval; OR, odds ratio; RAF, risk allele frequency; SNP, single nucleotide polymorphism.

^a^ Position is given with respect to genome build 37.

^b^ Risk allele/other allele
