# Supplementary material for: Fine-scale mapping of chromosome 9q22.33 identifies candidate causal variant in ovarian cancer
Source: PeerJ. 2024 Feb 14;12:e16918. doi: 10.7717/peerj.16918 (PMC10874173; doi:10.7717/peerj.16918)
Supplement: Supplemental Information 10 [file peerj-12-16918-s010.docx]

**Supplementary Table S8** The eQTL results of candidate causal variants from GTEx data.

| SNP | Position | Nearby genes and annotation | Gene | Tissue site | *P* |
| --- | --- | --- | --- | --- | --- |
| rs10988451 | 101741666 | intron variant of COL15A1 | COL15A1 | Nerve_Tibial | 3.07E-07 |
|  |  |  | COL15A1 | Cells_Cultured_fibroblasts | 1.08E-04 |
| rs7027650 | 101741969 | intron variant of COL15A1 | COL15A1 | Thyroid | 1.75E-04 |
|  |  |  | COL15A1 | Nerve_Tibial | 3.41E-07 |
|  |  |  | COL15A1 | Cells_Cultured_fibroblasts | 9.78E-05 |
| rs7021675 | 101752965 | intron variant of COL15A1 | COL15A1 | Nerve_Tibial | 1.20E-06 |
|  |  |  | COL15A1 | Cells_Cultured_fibroblasts | 1.77E-04 |
| rs4743305 | 101760026 | intron variant of COL15A1 | COL15A1 | Nerve_Tibial | 2.35E-06 |
| rs1889268 | 101767961 | intron variant of COL15A1 | COL15A1 | Nerve_Tibial | 1.33E-04 |
| rs7031588 | 101822302 | intron variant of COL15A1 | COL15A1 | Whole_Blood | 3.63E-05 |
|  |  |  | COL15A1 | Cells_EBV-transformed_lymphocytes | 2.32E-07 |
